# Identifying relevant diabetes and hypertension control management guidelines in primary healthcare and community settings in Indonesia: A Delphi survey

**DOI:** 10.1371/journal.pone.0310295

**Published:** 2024-11-27

**Authors:** Eti Poncorini Pamungkasari, Balgis Balgis, Jaap Koot, Jeanet Landsman, Zinzi Pardoel, Martin Rusnak, Dominika Plancikova, Victoria Sari, Stefanus Erdana Putra, Muhammad Hafizhan, Karina Fadillah Ahmad, Lely Pangesti, Ivan Sofian Wibowo, Ari Probandari

**Affiliations:** 1 Faculty of Medicine, Department of Public Health, Universitas Sebelas Maret, Surakarta, Indonesia; 2 Faculty of Medicine, Disease Control Research Group, Universitas Sebelas Maret, Surakarta, Indonesia; 3 Department of Health Sciences, Global Health Unit, University Medical Center Groningen, University of Groningen, Groningen, Netherlands; 4 Department Health Sciences, Applied Health Research Unit, University Medical Center Groningen, University of Groningen, Groningen, Netherlands; 5 Faculty of Health Care and Social Work, Department of Public Health, Trnava University, Trnava, Slovak Republic; CMC Vellore, IPH India, INDIA

## Abstract

The burden of non-communicable diseases (NCDs) in Indonesia is increasing, as evidenced by the latest Indonesian National Health Research, which shows an increase in diabetes prevalence, from 6.9% in 2013 to 10.9% in 2018, and hypertension, from 25.8% in 2013 to 34.1% in 2018. Hence, effective actions in community and primary health care (PHC) facility settings are necessary to tackle the burden of diabetes and hypertension, especially in low- and middle-income countries. The Indonesian government has issued numerous guidelines regarding NCDs. However, not all these guidelines can be applied to communities or PHCs. This study aimed to identify priority guidelines to support the community and PHC for NCD management using the Delphi survey method. These prioritized guidelines will serve as valuable resources for developing relevant, operational and comprehensive modules for community cadres and PHC staff involved in NCD management. The Delphi survey involved 25 experts and comprised three rounds using a questionnaire: 1) identification and assessment of guidelines, 2) assessment of the importance of guidelines, and 3) nomination of the three main priority guidelines. The results revealed three priority guidelines: NCD management guidelines, technical guidelines for *Pos Pembinaan Terpadu* (POSBINDU) NCDs, and integrated services for NCDs in PHC facilities. Additionally, priority guidelines were used to develop operational modules for community cadres and PHC staff in NCDs management. In conclusion, utilizing the Delphi method serves as a scientific approach to identify priority guidelines crucial for supporting the community and PHC in managing NCDs, particularly in countries with contexts similar to Indonesia.

## Introduction

Non-communicable diseases (NCDs) are the leading cause of death worldwide and one of the major health challenges of the 21st century [1]. According to the World Health Organization, NCDs account for 41 million deaths annually, comprising 74% of all deaths. To meet these targets, Indonesia has committed to preventing and treating NCDs. Prevention programs prioritize health promotion and understanding disease risks such as smoking, physical inactivity, unhealthy diet, and alcohol consumption [2]. Simultaneously, NCD treatment involves case finding and management.

The Indonesian National Health Research reported an increasing prevalence of NCDs in Indonesia in 2018 [3]. The prevalence of diabetes mellitus increased from 6.9% in 2013 to 10.9% in 2018 and 25.8% in 2013 to 34.1% in 2018 [3]. *Pos Pembinaan Terpadu* (POSBINDU) is a community-based program managed by non-medical volunteers (cadres) to screen and manage NCD risk factors. POSBINDU activities aim for early detection of NCD risk factors by screening for early follow-up of NCD risk factors through monitoring, education, and group activities to promote a healthy lifestyle [4]. Puskesmas (Public Health Centre or PHC) are first-line agents for the community to access the healthcare system [5].

Although the Indonesian government has published numerous NCD management guidelines, the performance of NCD screening activities is yet to be optimized [6]. This revealed missed opportunities in terms of the inputs, activities, and outputs of the POSBINDU implementation in screening for hypertension and its risk factors. Several contextual barriers were identified. The suboptimal coverage may be attributed to a lack of priority for NCD screening, awareness, access, and overlapping NCD-related programs. Suboptimal activities and reporting may stem from a lack of resources and limited time to undertake complex activities and reporting in accordance with the Ministry of Health guidelines [7]. Program implementers do not fully understand these targets. Additionally, the implementation did not follow technical instructions. Cadres have limited capabilities and require training or recruitment of new cadres [8]. The performance of NCD screening activities has yet to be optimized, even though the Indonesian government has published many NCD management guidelines. A previous study showed that the guidelines used in practice are fragmented and not applicable in PHC and community settings [4]. Guidelines were created for implementation in secondary and tertiary health facilities with complete facilities. However, these guidelines cannot be applied to PHC with limited resources. Moreover, the POSBINDU is run by cadres who are non-medical volunteers. The large number of guidelines makes it difficult to determine which ones should be applied. Hence, relevant and practical guidelines are essential to support PHC and POSBINDU in NCD management implementation for early detection, risk assessment, case finding, and referrals to reduce NCD-related morbidity and mortality [9]. This study aimed to identify priority guidelines to support the community and PHC for NCD management using the Delphi survey method. These prioritized guidelines will serve as valuable resources for developing relevant, operational and comprehensive modules for community cadres and PHC staff involved in NCD management.

## Materials and methods

The Delphi method using online questionnaires was used to identify the priority guidelines for NCD management by experts in Indonesia. This method has been used in various fields, including health research [10,11], and complies with the scientific rules that make it reproducible and applicable to other systems. The Delphi method was chosen because of the ease of submitting online questionnaires within a relatively short survey period.

**Fig 1:**
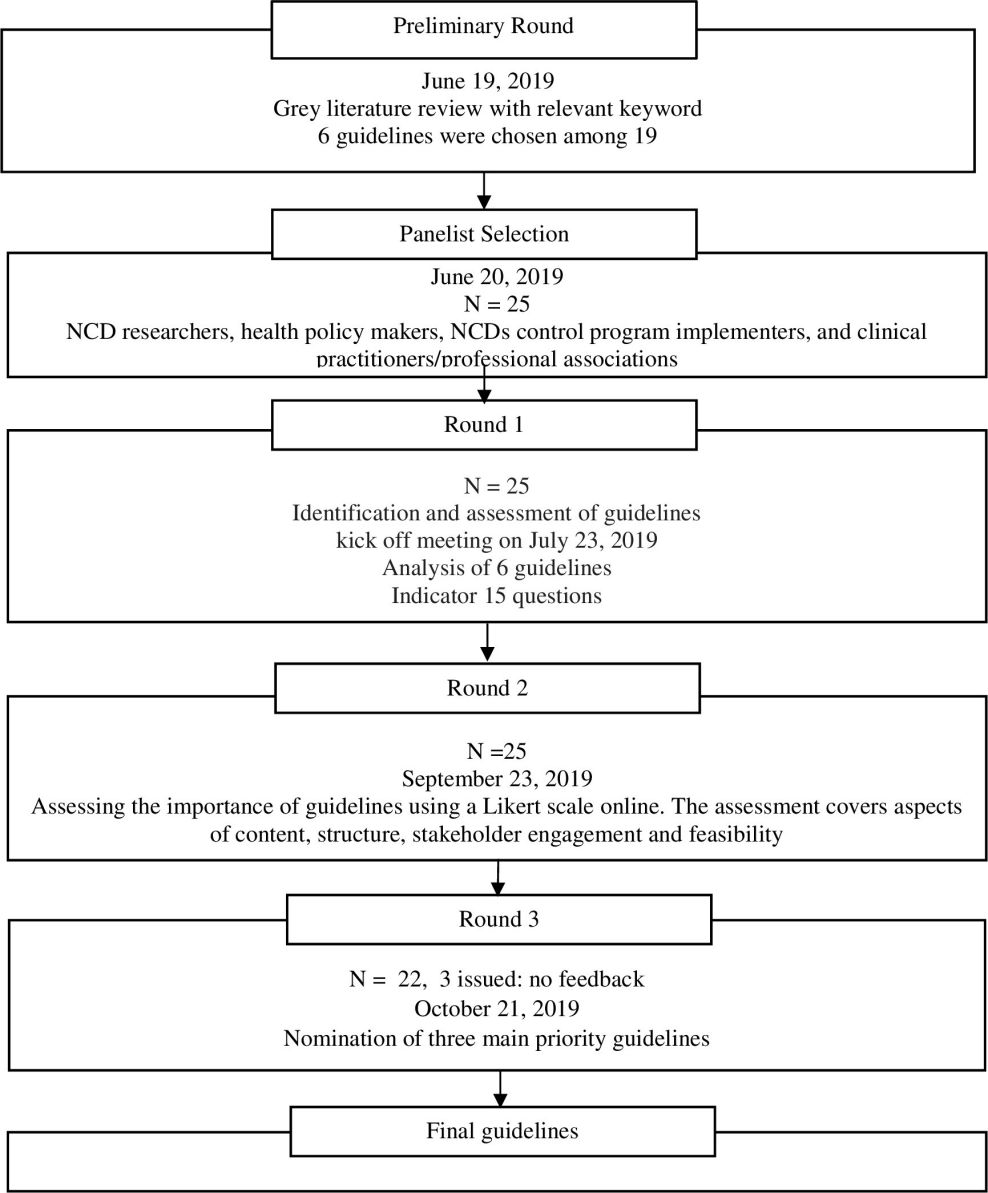
Flowchart study design.

The Delphi survey was conducted in three rounds [10]: 1) identifying and assessing guidelines, 2) assessing the importance of guidelines, and 3) nominating the three most suitable guidelines (Fig 1). In our study, consensus mainly pertained to developing operational guidelines for screening and case management of NCDs in PHC facilities and communities.

This study met all applicable standards for ethics of research integrity. The participants signed a consent form sent by e-mail, and the data were stored on the drive. Minors were not included in this study. Ethical approval was granted by the Health Research Ethics Board of Universitas Gadjah Mada (number: KE/0487/04/2019).

### Preliminary round

We conducted literature reviews that included guidelines in the form of consensus, reporting, and stakeholder presentations used as guidelines in the field [12]. Not all guidelines in Bahasa are in the form of books or articles published by official publishers regarding the management of NCDs, especially diabetes mellitus and hypertension. We used a web browser search engine with relevant keywords such as the POSBINDU guidelines, hypertension management guidelines, diabetes mellitus management guidelines, chronic disease management programs, integrated NCDs services, and integrated elderly health service posts. The selection criteria were launched before 2014 to keep them relevant and have been implemented in Indonesia.

We prepared questionnaires for round 1 based on the identification and description of the guidelines for NCD management. Subsequently, 25 experts assessed whether the selected list of guidelines was relevant to the NCDs management program. Additionally, issues were grouped to prevent duplication. The questionnaire is presented in Table 1.

**Table 1:** Questions for Delphi survey round 1.

Number	Questions
1	Are the following points clearly stated? • The group of patients • Health problems • Care provider • Care setting
2	Are all important options and outcomes clearly defined?
3	Were explicit and reasonable processes used to identify, select, and combine evidence?
4	Are explicit and reasonable processes used to consider the relative value of different outcomes? (consider who participates in this process and whose values are considered)
5	Are the guidelines resistant to clinically plausible practice variations?
6	Had been all potential stakeholders involved? (consider benefits, risks, costs)?
7	Are the guidelines based on the latest data, that is, how up-to-date is the guideline? (consider: date of publication of the most recent evidence considered, date on which final recommendations were made, and suggested date for review)
8	Have the guidelines been reviewed and tested?
9	Was there a conflict of interest in the development and publication of this guide? (consider how independent the individual who developed this guide was?)
10	Are practical and clinically important recommendations made?
11	How strong are the recommendations? (consider how strong the evidence is on which the guidelines are based)
12	Do these recommendations apply to your patients?
13	Do the guidelines offer an opportunity for significant improvement in the quality of care? (Consider: Is there large variation or uncertainty in current practice? Does it contain evidence that could have an important impact on management? Does it affect many patients, or concerns patients at high risk, or involves high costs, that even small changes in practice can have a big impact?)
14	What are the obstacles of implementation?
15	Can these obstacles be overcome?

### Panelist selection

First, we selected a panel consisting of stakeholders, subject experts, and facilitators. The experts involved in the Delphi survey had to comply with the inclusion criteria: have knowledge and experience in diabetes mellitus and hypertension screening and management in community and PHC settings, be willing to participate, and have sufficient time. We excluded experts who could not be contacted because they did not have contact details such as e-mail or telephone numbers, provided late responses, or could not complete the questionnaire [13].

A total of 25 participants, including researchers, policymakers, program implementers, and clinical practitioners or professional associations, were selected as prospective representatives. Panelists were identified based on recommendations from the Ministry of Health Indonesia, Indonesian Health Research and Development Agency, Provincial Health Office, District Health Office, general practitioners involved in NCD interventions, non-governmental organizations, and academics. We explained the purpose of the study, described the procedures, the expected duration, and the voluntary nature of the panelists’ decisions in the introduction of the Delphi consensus meeting. The panelists’ backgrounds are presented in Table 2.

**Table 2:** Background information of panelists.

Name	Role	Level
Panelist 1	Directorate of NCDs Prevention and Control, MoH	National
Panelist 2	Staff of NCDs Prevention	Province
Panelist 3	Staff of NCDs Prevention	Province
Panelist 4	Staff of NCDs Prevention Health Department	District
Panelist 5	Staff of NCDs Prevention Public Health Center	District
Panelist 6	Staff of NCDs Prevention Public Health Center	District
Panelist 7	Staff of NCDs Prevention Health Department	District
Panelist 8	Staff of NCDs Prevention Program Health Department	District
Panelist 9	Sub-Office Head of NCDs Prevention Health Department	District
Panelist 10	Staff of NCDs Prevention Public Health Center	District
Panelist 11	Staff of NCDs Prevention Public Health Center	District
Panelist 12	Coordinator of NCDs Prevention Health Department	District
Panelist 13	Social Security Agency of Health	District
Panelist 14	Advisory Board of SUNI-SEA project Indonesia	University
Panelist 15	Staff of NCDs Prevention Program	Province
Panelist 16	Advisory Board Partner of SUNI-SEA	University
Panelist 17	Representative of Indonesian Association of Clinics and Health Care Facilities	District
Panelist 18	Representative of Indonesian Association of Clinics and Health Care Facilities	District
Panelist 19	Representative of Indonesian Association of Clinics and Health Care Facilities	District
Panelist 20	Advisory Board Partner of SUNI-SEA, a general practitioner	University
Panelist 21	Representative of Indonesian Association of Clinics and Health Care Facilities	District
Panelist 22	Internal Medicine Specialist	Province Hospital
Panelist 23	Endocrinologist	Province Hospital
Panelist 24	Section Head of NCDs Prevention	Province
Panelist 25	Staff of NCDs Prevention Public Health Center	District

### Round 1

The Delphi survey round 1 was conducted during the kick-off meeting on July 23, 2019, using a questionnaire comprising 15 questions. The questionnaire was designed to identify and describe gaps in the application of each guideline for NCD management. The questionnaire for round 1 was adapted from “Critical Appraisal Checklist for An Article on Guidelines” from the Department of General Practice University of Glasgow. The questionnaire was then translated into Indonesian and a preliminary study was held to validate the translation content. Panelists were asked to identify and assess the relevance of the preselected guidelines for the management of NCDs. Each panelist answered each question on the checklist with a choice of "yes" or "no" or "don’t know" answers. After the questionnaire filling process was complete, we counted the proportion of “yes” answers for each question.

### Round 2

The round 2 Delphi survey was conducted via e-mail on September 23, 2019. During this round, the panelists were asked to rate the importance of the preselected guidelines using a Likert scale. The answer categories were “slightly important,” “moderately important,” “important,” or “very important.” For each guideline, four indicators were assessed: content, structure, stakeholder involvement, and implementation feasibility. The results from all panelists are displayed as percentages sorted by importance. Panelists were given 1 week to complete round 2. The questionnaire link was sent via e-mail. If any panelists encountered difficulty and did not fill out the questionnaire, they would be followed up by telephone to determine the reason for not completing it.

### Round 3

Round 3 was conducted online via e-mail on October 21, 2019. All panelists were asked to nominate three main priority guidelines for managing NCDs in community and PHC facilities, especially diabetes mellitus and hypertension, in Indonesia for the next 10 years, as well as explain the reasons for their choices. The results of the panelists’ choices were assessed based on the percentage of each guideline; the three guidelines with the highest percentage scores were selected as the top priority. We decided on three priority guidelines to identify the strengths and weaknesses of each. Besides, we also added the open question at the end of the questionnaire to understand the reasons of the panelists in nominating the best guidelines on their perspectives.

### Analysis

After completing the panel process, we performed descriptive-quantitative data analyses on the ranking of each guideline based on expert perceptions. Data analysis was conducted directly after each round to avoid inaccuracies and misunderstandings regarding the topic of discussion. Descriptive-quantitative data analyses were performed to evaluate consensus agreement [14]. We compared the responses from each panelist and reported the results as a percentage. The conclusion for guideline priority choosing was then determined by the highest percentage of panelist’s responses.

## Results

### Preliminary round

In the literature review, we found 19 guidelines, of which six were appropriate for the chosen topic and fulfilled the inclusion criteria. Additionally, we verified the results of our grey literature review by sending the preliminary round results to all chosen panelists 1 week before the kick-off meeting and asking for their agreement during the meeting, thus ensuring that no important documentation had been left out. The selected guidelines are as follows (See Table 3).

1. NCDs management guideline;
2. Integrated services for NCDs in primary healthcare facilities;
3. Technical guidelines for POSBINDU of NCDs;
4. POSBINDU cadre pocket book;
5. Management of hypertension prevention and monitoring programs
6. Consensus on management of hypertension 2019

**Table 3:** The selected guidelines.

No	Guideline and Year of Publication	Authors/ Publisher	Content	Target Group
1	Non-Communicable Diseases Management Guideline 2019	Ministry of Health of the Republic of Indonesia	Monitoring and evaluation form of prevention and controlling of NCDs, recapitulation of NCDs cases form	Health workers, cadres, volunteers
2	Integrated Services for Non-Communicable Diseases in Primary Healthcare Facilities 2019	Provincial Health Office	Management of service programs at health facilities related to NCDs	Health workers
3	Technical Guidelines for POSBINDU of Non-Communicable Diseases 2016	Ministry of Health of the Republic of Indonesia	Criteria for controlling NCDs risk factors, frequency and period of monitoring of NCDs risk factors (Ministry of Health Indonesia)	Health workers, cadres, volunteers
4	POSBINDU Cadre Pocket Book 2019	Ministry of Health of the Republic of Indonesia	Guidelines and technical instructions for implementing POSBINDU activities	Cadres
5	Management of Hypertension Prevention and Monitoring Programs 2018	Ministry of Health of the Republic of Indonesia	Hypertension prevention and control program management and calculation of the achievement of patients with hypertension	Health workers
6	Consensus on Management of Hypertension 2019	Indonesian Society of Hypertension	Agreement on the principles of hypertension management	Health workers (cardiologist, neurologist nephrologist)

### Rounds 1

The results for round 1 are presented in Table 4. All panelists agreed that the guidelines already addressed and covered health issues describing patient grouping and treatment, resistance to clinically reasonable practice variations, making practical and clinically important recommendations, and overcoming barriers to implementation.

**Table 4:** Results of round 1 of the Delphi survey from 25 experts.

Theme	Question	1^st^ Guideline n (%)	2^nd^ Guideline n (%)	3^rd^ Guideline n (%)	4^th^ Guideline n (%)	5^th^ Guideline n (%)	6^th^ Guideline n(%)
Clarity	Patient group	25 (100)	21 (84)	25 (100)	23 (92)	25 (100)	21 (84)
	Health problem	25 (100)	24 (96)	25 (100)	23 (92)	25 (100)	23 (92)
	Care provider	25 (100)	23 (92)	25 (100)	21 (84)	25 (100)	19 (76)
	Care setting	23 (92)	22 (88)	25 (100)	20 (80)	23 (92)	20 (80)
Important options and outcomes		24 (96)	24 (96)	24 (96)	24 (96)	25 (100)	25 (100)
Explicit and sensible process	to identify, select and combine evidence	20 (80)	19 (76)	20 (80)	20 (80)	23 (92)	21 (84)
Explicit and sensible process to consider the relative value of different outcome	Participant	16 (64)	15 (60)	20 (80)	16 (64)	19 (76)	24 (96)
	Values	18 (72)	16 (64)	22 (88)	17 (68)	17 (68)	23 (92)
Resistance of sensible variations clinically		22 (88)	19 (76)	23 (92)	24 (96)	23 (92)	21 (84)
Potential stakeholders’ involvement		18 (72)	17 (68)	22 (88)	17 (68)	20 (80)	19 (76)
Benefit		18 (72)	17 (68)	22 (88)	17 (68)	20 (80)	19 (76)
Risk		18 (72)	18 (72)	19 (76)	17 (68)	21 (84)	19 (76)
Cost		18 (72)	15 (60)	20 (80)	14 (56)	20 (80)	16 (64)
Update	The publication date	18 (72)	16 (64)	15 (60)	16 (64)	`13 (52)	18 (72)
	The final recommendation date was made	14 (56)	12 (48)	15 (60	13 (52)	11 (44)	16 (64)
	The review date suggested	13 (52)	11 (44)	13 (52)	9 (36)	10 (40)	14 (56)
Peer reviewed and tested guideline		11 (44)	11 (44)	10 (40)	10 (40)	10 (40)	10 (40)
Conflict of interest	independent author	7 (28)	6 (24)	7 (28)	8 (32)	5 (20)	5 (20)
Recommendation	Practical and important	20 (80)	17 (68)	19 (76)	22 (88)	22 (88)	21 (84)
	Strength	15 (60)	14 (56)	18 (72)	15 (60)	18 (72)	23 (92)
	Applicability	24 (92)	24 (96)	23 (92)	24 (96)	23 (92)	23 (92)
Offering improvement in the quality of care	Variation or uncertainty	16 (64)	18 (72)	18 (72)	18 (72)	19 (76)	19 (76)
	Evidence of important impact on management	23 (92)	22 (88)	22 (88)	22 (88)	23 (92)	23 (92)
	Cost-effectiveness	19 (76)	18 (72)	20 (80)	20 (80)	21 (84)	21 (84)
Barriers of implementation		19 (76)	19 (76)	20 (80)	20 (80)	21 (84)	20 (80)
Solution of barriers		22 (88)	22 (88)	22 (88)	22 (88)	23 (92)	23 (92)

The panelist considered that the first, third, and fifth guideline showed clarity for all questions. All panelists also agreed that the fifth and sixth guidelines provide the most important options and outcomes. Around 92% of the panelists argued that the fifth guideline had explicit and sensible process. However, when the relative value of different outcomes was taken into account, the 6th guideline was considered better compared to the 5th guideline.

More than 90% of the panelists agreed that the third, fourth, and fifth guideline had better resistance to sensible variations clinically compared to the other three guidelines. The third guideline had the greatest potential stakeholder involvement, while the fifth guideline provided the most benefit with the least risk and cost. The sixth guideline was also the most recent guideline in all criteria.

Less than 50% of the panelists also argued that all guidelines were poorly reviewed and tested. Furthermore, only around 20–32% of the panelists suggested that all guidelines were free from conflict of interest. The fourth and fifth guideline provided us with excellent practical and important recommendations. Strongest recommendation was provided by the 6th guideline, while the second and fourth guideline provided the most applicable recommendation.

Meanwhile, almost all of the panelists agreed that the fifth and sixth guideline offered improvement of the care quality better than the other guidelines. The fifth guideline showed the greatest barriers to be implemented. However, this guideline also provided the solution for those barriers.

### Rounds 2

The results for round 2 are presented in Table 5. The results showed that most panelists classified all guidelines as “very important” for each question indicator. All 25 panelists gave feedback to our email.

**Table 5:** Results of round 2 of the Delphi survey from 25 experts.

Guideline	Indicator	A Little Important n(%)	Quite Important n(%)	Important n(%)	Very Important n(%)
1^st^ Guideline	Content	0 (0)	2 (8)	12 (48)	11 (44)
	Structure	0 (0)	4 (16)	16 (64)	5 (20)
	Stakeholder Involvement	0 (0)	4 (16)	10 (40)	11 (44)
	Easiness of Implementation	0 (0)	1 (4)	15 (60)	9 (36)
2^nd^ Guideline	Content	1 (4)	2 (8)	13 (52)	9 (36)
	Structure	2 (8)	3 (12)	16 (64)	4 (16)
	Stakeholder Involvement	1 (4)	3 (12)	8 (32)	13 (52)
	Easiness of Implementation	0 (0)	2 (8)	13 (52)	10 (40)
3^rd^ Guideline	Content	0 (0)	3 (12)	14 (56)	8 (32)
	Structure	1 (4)	5 (20)	15 (60)	4 (16)
	Stakeholder Involvement	1 (4)	3 (12)	14 (56)	7 (28)
	Easiness of Implementation	1 (4)	2 (8)	15 (60)	7 (28)
4^th^ Guideline	Content	0 (0)	5 (20)	9 (36)	11 (44)
	Structure	3 (12)	4 (16)	11 (44)	7 (28)
	Stakeholder Involvement	2 (8)	3 (12)	10 (40)	10 (40)
	Easiness of Implementation	1 (4)	3 (12)	10 (40)	11 (44)
5^th^ Guideline	Content	1 (4)	1 (4)	17 (68)	6 (24)
	Structure	1 (4)	3 (12)	17 (68)	4 (16)
	Stakeholder Involvement	0 (0)	3 (12)	15 (60)	7 (28)
	Easiness of Implementation	0 (0)	2 (8)	11 (44)	12 (48)
6^th^ Guideline	Content	1 (4)	1 (4)	14 (56)	9 (36)
	Structure	1 (4)	2 (8)	18 (72)	4 (16)
	Stakeholder Involvement	1 (4)	4 (16)	14 (56)	6 (24)
	Easiness of Implementation	1 (4)	1 (4)	13 (52)	10 (40)

Most of the panelists rated that the first and second guidelines were "important" in three indicators, i.e. content, structure, and easiness of implementation. They were also "very important" in the stakeholder involvement indicator. Besides, more than half of the panelists voted "important" in all indicators of the third and sixth guidelines. Meanwhile, the fourth guideline was rated "very important" in two indicators (content and easiness of implementation), "important" in one indicator (structure), and tied between "important" and "very important" in one indicator (stakeholder involvement). Lastly, the 5th guideline was rated "important" in three indicators (content, structure, and stakeholder involvement) by the majority of the panelists, and rated "very important" for easiness of implementation.

### Rounds 3

**Fig 2:**
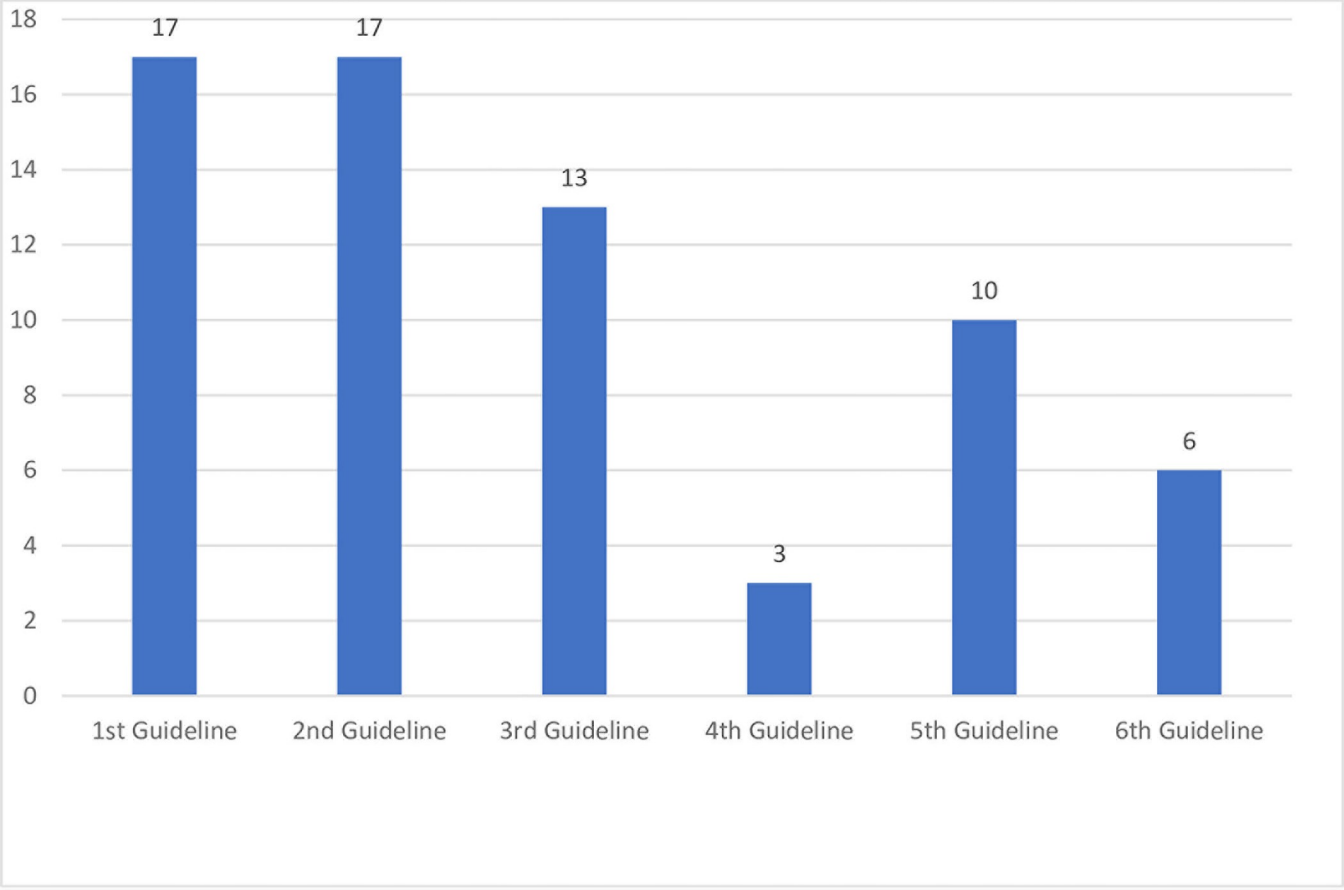
Votes for the priority of guidelines.

The result of round 3 is shown in Fig 2. In round 3, the first and second guidelines were the most chosen by the panelists, each by 17 (77%) panelists. The third, fourth, fifth, and sixth guidelines were selected by 13 (59%), 3 (14%), 10 (45%), and 6 (27%) panelists, respectively. During this round, 3 panelists did not complete the questionnaire and did not answer the phone call. The three guidelines with the highest score were the Non-Communicable Diseases Management Guideline 2019 from the Ministry of Health of the Republic of Indonesia, the Integrated Services for Non-Communicable Diseases in Primary Healthcare Facilities Guideline 2019 from the Provincial Health Office, and the Technical Guidelines for POSBINDU of Non-Communicable Diseases 2016 from Ministry of Health of the Republic of Indonesia. The panelists who chose the first guideline argued that it featured comprehensive coverage, discussing policies, targets, achievement indicators, strategies, and management. They believed it provided clear and easily understandable guidelines and recommendations, facilitating practical application. The second guideline, as assessed by the panelists, aligns with the primary duties and functions of health workers in PHC. It can be used as a guide for implementing activities within this context, serving as an indicator for achieving minimum service standards at the district or city level. Additionally, it is well suited for the integrated management of NCDs and can be seamlessly integrated with other PHC activities. The third guideline was selected because it describes the technical implementation of the NCDs POSBINDU and can be directly applied in the field.

## Discussion

This study demonstrates that the Delphi method can be implemented to systematically determine program priorities and can be scientifically justified. The Delphi method is widely used to build guidelines systematically based on experts [14]. For example, this method was used to develop global guidance for the recognition of Tumor-induced Osteomalacia [15]. On the other hand, a consensus was developed using the Delphi survey to recognize acute appendicitis in children [16]. We used the same Delphi method as the one conducted by others [15,16], which consisted of a preliminary round and 3 main rounds, involving a panel of experts who are competent in the relevant field of research, including those with experience handling relevant cases. In both studies, before conducting the three-round Delphi survey, a literature search was conducted using specific criteria, and articles were selected based on content review. In Round 1, panelists were asked to rate their agreement with recommendation statements using a five-point scale (strongly disagree, disagree, neither agree nor disagree, agree, or strongly agree), panelists also could suggest new features for the next round [15,16]. In Rounds 2 and 3, results from the previous round were presented, and panelists were asked to reassess the importance of selected features by indicating if they considered each feature important (yes/no) [16]. Recommendations deemed important or very important by ≥70% of participants were included on the consensus list after each round [15,16].

Relevant to the other study, we conducted a three-round Delphi survey preceded by literature reviews. This study involved panel members who are experts in the management of diabetes mellitus and hypertension in community and primary healthcare settings. In this Delphi survey, we identified and assessed the relevance of guidelines to NCD management in the field in the first round, then evaluated their importance in the second round using a Likert scale. In the third round, we nominated the three most suitable guidelines. Consistent with previous studies, we presented results from each preceding round as considerations for subsequent rounds. In contrast to the other study, there was no guideline elimination after each round. Besides, in Round 3, when prioritizing guidelines, panelists provided feedback explaining their choices for each selected guideline.

In the first round, the third guideline got the highest score, followed by the sixth and fifth guidelines respectively. The three guidelines are superior to other guidelines, for example in terms of better evidence and stronger recommendations. They also have good clarity, stakeholder involvement, and explicitly explain the process of compiling and searching for evidence compared to other guidelines. Meanwhile, in the second round, the first, fifth, and sixth guidelines obtained the highest scores. Panelists rated the three guidelines as ’important’ and ’very important’ based on content, structure, and ease of implementation. However, in the third round, the panelists selected the first, second, and third guidelines as the most appropriate for implementation in community and PHC settings. The fifth and sixth guidelines were not selected despite having high scores in rounds 1 and 2 because they are more suitable for implementation in secondary and tertiary healthcare facilities. Meanwhile, the fourth guideline is intended for community healthcare settings, but this guideline has lower quality and importance than the other guidelines.

The three guidelines with the highest scores were noncommunicable disease management, integrated services for NCDs in primary healthcare facilities, and technical guidelines for POSBINDU. Guidelines that are easy to understand are more likely to be used [17]. Simple and plain language strengthens perceived behavioral control, creates more positive attitudes toward using guidelines, and results in stronger behavioral intentions to implement these guidelines [18].

Improvements in implementing NCD treatments, from guidelines to practices, are necessary. Not all the guidelines are easy for cadres to use in practice. Previous research in Indonesia showed a lack of human resources for hypertension screening in terms of capacity and quantity [6]. As cadres often handle several programs simultaneously, they require easily implementable guidance. Additionally, this research found a lack of competency among cadres in screening examinations, necessitating practical guidance and training. The ability of cadres to provide health education is still lacking, highlighting the need for guidelines that provide simple and complete education for the community.

The first selected guideline, Non-Communicable Diseases Management Guideline, gives a big picture of why POSBINDU needs to be held. This guideline explains POSBINDU’s objectives, goals, types of activities carried out, and how it is monitored. The second selected guideline, Integrated services for NCDs in PHC facilities, were chosen because it provides technical explanations on how to carry out certain procedures such as history taking, blood pressure checks, blood glucose checks, and anthropometric examinations. This guideline allows non-health worker cadres to carry out those simple procedures during the POSBINDU. Meanwhile the third selected guideline, Technical Guidelines for POSBINDU of Non-Communicable Diseases, was chosen because it explains how POSBINDU should be implemented. This guideline provides a detailed explanation regarding the amount of personnel and tools required, as well as the sequence of POSBINDU activities from participant registration to the end. A study showed that to become successful, a guideline targeting health professionals must achieve a balance between standardized practice and flexibility in clinical judgment [19]. Excessive flexibility compromises quality standards, while rigid procedures hinder professional autonomy. Sometimes, health professionals encounter complex cases, necessitating a tailor-made approach based on their expertise [20]. However, this is not the case with this guideline. The end-user targets were PHC staff and POSBINDU cadres. POSBINDU cadres are non-medical volunteers from various backgrounds, lacking expertise in either clinical practice or the public health field; therefore, detailed technical instructions ready to be applied are required [21].

First guideline explains the objectives of the program, while second guideline explains how to do simple clinical examinations. Then the third guideline explains how POSBINDU should be held. Among many guidelines regarding NCDs, the panelists have chosen these three guidelines. We felt it necessary to develop a new guideline that combines essential parts of the three guidelines. This will make it easier for cadres to carry out the POSBINDU. Our study yields expert-agreed priority guidelines for further use at the community level. Existing guidelines often encounter barriers related to time allocation and translation [22]. Involving stakeholders in determining priority guidelines using the Delphi method can help reduce these barriers [22]. These priority guidelines were adopted to develop practical materials or operational modules for community health cadres and health workers to screen for NCDs. These modules can be adapted to societal contexts to address issues like stigma. These materials have been promoted by the Ministry of Health to fill the gap in the implementation of NCD screening in Indonesia. The Delphi method used in this study can be an example of a scientific approach for identifying relevant priority guidelines to support NCD management in the community and PHC, particularly in countries with similar contexts as Indonesia.

The panelists selected three guidelines based on their relevance and current applicability in the community and PHC settings. This study underscores the need to refine the guidelines for the operational tools readily employed by healthcare cadres in Indonesia. Given the diverse levels of knowledge and skills among cadres, a guide accommodating these discrepancies is urgently needed. Scaling up NCD interventions in South-East Asia (SUNI-SEA) has addressed this need by developing operational modules promoted by the Indonesian Ministry of Health.

### Strengths and limitations

This study is the first Delphi survey to review the NCDs management guidelines for PHC and community settings in Indonesia. The strength of this study is that the panelists represented perspectives related to NCDs, such as policymakers, program implementers, and clinical practitioners. The limitation of this study is that only stakeholders were involved in determining the priorities of the guidelines. Involving target audiences such as cadres and health workers may be important in determining the applicability of these guidelines. In the future studies relevant to the PHC and POSBINDU settings, health cadres and health workers should be included to prove the most relevant input on how the available guidelines aid or impede their activities. In addition, the input from health cadres and health workers on future guidelines’ content should be laid out are essentials.

## Conclusion

This three-round Delphi survey concluded with three selected guidelines relevant to developing operational modules to support NCD management in the community and PHC in Indonesia. The NCD-relevant guidelines published in Indonesia contain fragmented content; one guideline can explain the indicators of the program, while others explain the clinical aspects of NCD management. By developing a comprehensive operational module based on these guidelines and engaging stakeholders in the decision-making process, the SUNI-SEA aims to bridge the gap in NCD screening implementation and support community health efforts. Utilizing the Delphi method serves as a scientific approach to identify priority guidelines crucial for supporting the community and PHC in managing NCDs, particularly in countries with contexts similar to Indonesia.

## Supporting information

S1 File Human subject research checklist. (DOCX)
